# Some Observations on Military Surgery during One Year’s Service in the 23rd General Hospital, B. E. F., France

**Published:** 1918-03

**Authors:** 


**Some Observations on Military Surgery During One Year's Service in the 23rd General Hospital, B. E. F., France.** By JAMES NEFF, Hon. Lieut.-Col., R. A. M. C., and JOHN G. O'MALLEY, Hon. Lieut., R. A. M. C. Abs. from *Surgery, Gynecology, and Obstetrics,* January, 1918.

After enumerating some of the common difficulties of military surgery, and the methods of handling the wounded, the authors proceed to discuss the treatment of infected wounds.

When particles of missile and clothing have been removed and the abcess or infected region opened up, they recommend drainage by fenestrated rubber tubes. They believe that all irrigations are superfluous and that they uselessly cause pain. Their only real service is that of a deodorant.

The authors record their observations of the use of hypochlorite solutions. During the year, in a hospital of 1500 beds, more than 7000 surgical cases were treated. The hospital was divided into three services. During the first six months eusol was used in one of the services, by the method of intermittent irrigation through multiple tubes. The same method was employed in about half the cases of a second service. It was not used at all, however, in the third service. In the second half year it was again used in the first service and not at all in the third. As the surgical cases were evenly distributed among the services, an excellent opportunity was afforded for watching the effects of the treatments with and without the hypochlorous acid solution. The authors state without hesitation that in no single case did they observe any of the phenomenal results so often claimed by the advocates of the irrigation method. They are convinced that the old established treatment of incision and adequate drainage gives as good if not better results. They furthermore declare that in not a single severely infected case did they see any hastening in the process of healing that could be directly attributed to the use of hypochlorous acid solutions.

They then proceed to attack the Carrel-Dakin theory of treatment on the ground of its complexity and strict requirements. They do not believe that their bad results from its use can fairly be attributed to a failure to carry out the standardized formula of application. They admit that if all the requirements of the technique could actually be carried out complete sterilization might be obtained; but in the light of their own experience they have come to believe that the complete sterilization of a septic wound by means of any solution whatever is too absurd to consider seriously.

Although it is certain that bacteria on the surface of the wound are washed away by the irrigation, there is no means of reaching those in the tissues which are the real cause of the infected condition. The Carrel-Dakin method, therefore, must be regarded merely as an adequate means of drainage, but in this connection it is well to remember that years ago prominent surgeons demonstrated that all irrigations of infected wounds were useless.

Reasons for the failure of antiseptics are set forth as follows:

1. It is impossible by any method of drainage or the use of any number of tubes to reach the pathogenic organisms in the tissues.

2. Micro-organisms on the surface of a wound are harmless. They have been thrown out of the tissues by exuding serum, and it is obviously useless merely to wash these away.

3. The difficulty of reaching organisms in the tissues by any method of irrigation is increased by the fact of the flow of serum outward under pressure, thus completely preventing any progress from the surface of an irrigating fluid upward into the tissues. Such absorption as takes place is through the superficial lymphatic vessels only, and substances so taken up do not reach the intervascular spaces. If any fluid is to be used to relieve pain and to assist drainage, hot water is as good as any.

The authors believe that vaccine treatment for infected wounds in the base hospitals has but little value, because cases are acute and do not remain long enough for beneficial results to be obtained.

Advice is given as to the treatment of various types of war wounds:

*Wounds of the Head.* — All missile wounds of the head should be operated unless it is clear that the skull has not been injured. Foreign material, depressed fragments of bone, and necrotic tissues should be removed with as little disturbance of tissues as possible and with the smallest possible opening of the skull. It is not advisable, however, to remove shrapnel and bullets from the brain unless they are accurately localized near the surface and easily accessible. When the fragments are too deep for removal and signs of infection develop, a tube of rubber, aluminum, or glass should be inserted as near as possible to the metal. Operations to relieve pressure from hemorrhage are usually unsatisfactory, but should be performed with the smallest possible opening in the bone, provided paralysis of the basic centers has not set in.

*Wounds of the Neck.* — When the important structures of the neck have been injured the greatest care must be exercised. The operator must be on his guard against emphysema of the subcutaneous tissues and edema of the glottis, when the trachea or larynx is injured : cellulitis is also to be feared in injuries of the pharynx and esophagus. The suture of injured vessels in this region is often of utmost importance in preventing fatal hemorrhage.

*Wounds of the Chest.* — Hemothorax is usually caused by a wound in the lung. So also is hemoptysis, although the latter may be caused merely by concussion. In cases of hemothorax it has been found good practice, after the hemorrhage has ceased, to aspirate from one fourth to one half of the blood in the pleural cavity. Gas infection in the cases of hemothorax is usually indicated by a sudden rise of temperature, prostration, pallor, and rapid pulse and respiration. Immediate drainage of the pleural cavity is imperative. The physical signs are those of a hemo-pneumo-thorax.

*Wounds of the Abdomen.* — The authors have found wounds of the small intestine more serious than those of the large. Drainage of the peritoneum is absolutely necessary. Liver wounds are especially serious because of possible complications. Wounds of the bladder call for continuous drainage, suprapubically, perineally, or *per urethram*, as may be indicated.

*Wounds of the Rectum.* — In case of extensive wounds of the rectum, colostomy should always be performed.

*Wounds of the Joints.* — When there is no infection, as in the case of most bullet wounds, aspiration of the blood, rest, and extension, with passive and active motions as soon as condition will permit, are indicated. When the wound is infected, as in the case of most shrapnel wounds, suppurative arthritis almost always occurs. The removal of foreign bodies and free drainage should be effected. In case of severe infection of the knee-joint the patella may be raised by means of a horse-shoe incision. The joint may be kept open in this manner until the infection has subsided. The patella is then removed and the joint allowed to ankylose. In other joints, especially the elbow, resection is often necessary. Later, bone transplantation may be performed in such cases. Such radical measures are not called for except in cases of severe infection.

*Infected Compound Fractures.* — The authors report but little progress in dealing with this most difficult surgical problem. Immobilization in a proper position and free drainage are the only sure methods. In cases of extensive comminution with severe or persistent infection, they believe that the removal of the splinters and squaring of the bones with, later, bone transplantation may be good practice; but they think that this radical operation is resorted to more frequently than is good for a functional result, especially by the French military surgeons.

*Amputations.* — In cases where the damage to a limb is so great, or the infection so virulent, that the usefulness of the limb, if saved, is doubtful, the authors recommend amputation. This applies particularly to gas infections and to cases in which there have been more than one secondary hemorrhage in a septic field. The work should be done as quickly as possible, very little anesthetic used, and the stump left wide open. Unless the injury is low down in the leg, it is best to amputate above the knee.

*Wounds of the Spinal Column.* — There is little hope for gunshot wounds of the column, even if they do not prove immediately fatal. In cases where the cord itself has actually been injured, operations are usually of little avail, but they should be performed with the hope that in some cases the paralysis is due to pressure only.

*Wounds of Blood-Vessels.* — The authors find that blood-vessel suture plays but a small part in war surgery, because of the impossibility of suture in an infected field. Where large arteries are ligated, especially in gas infection, there is always danger of secondary hemorrhage, which is greater the nearer the ligation is to the heart. They make the following recommendation in case of secondary hemorrhage in infected fields : " If secondary hemorrhage occurs more than once, waste no time in further ligations but amputate immediately. If this rule is followed many lives will be saved. "

*Wounds of the Nerves.* — These are not as common as might be supposed. Where a lesion exists, the wound should be allowed to heal completely before an operation is performed. The operation consists in a resection of the bulbous ends and suture of the stumps. When the nerve has been imbedded in fibrous tissue, it should be freed and surrounded by fat or muscle.

